# 43. Impact of a Five-Year Intervention of an Antimicrobial Stewardship Program on the Optimal Antibiotic Prophylaxis Selection in Surgery in a Hospital without Restrictions on Antibiotics Prescription in Costa Rica

**DOI:** 10.1093/ofid/ofab466.245

**Published:** 2021-12-04

**Authors:** José P Díaz-Madriz, Esteban Zavaleta-Monestel, Jorge A Villalobos-Madriz, Alison V Meléndez-Alfaro, Priscilla Castrillo-Portillo, Ana F Vásquez-Mendoza, Gabriel Muñoz-Gutierrez, Sebastián Arguedas-Chacón

**Affiliations:** 1 Hospital Clínica Bíblica, San José, San Jose, Costa Rica; 2 Universidad de Ciencias Médicas (UCIMED), San José, San Jose, Costa Rica; 3 Universidad de Costa Rica, San José, San Jose, Costa Rica

## Abstract

**Background:**

In a private hospital without restrictions on antibiotic prescription, the success of an Antimicrobial Stewardship Program (ASP) depends mainly on prospective feedback and education. Previously, the ASP of this hospital (PROA-HCB) managed to achieve a positive impact on the antibiotic prophylaxis in cesarean delivery. The purpose of this study is to characterize the impact after implementing the PROA-HCB on the optimal prophylaxis selection of all the procedures included in the clinical guideline for surgical antibiotic prophylaxis in adult patients.

**Methods:**

A retrospective observational study that compares the selection, duration, antibiotic consumption, bacterial resistance profiles and patient’s safety outcomes regarding antibiotic use for all surgical prophylaxis prescription over six months for the periods before (pre-ASP) and after a five-year intervention of PROA-HCB (post-ASP).

**Results:**

After a five-year intervention, the percentage of optimal selection of antibiotic prophylaxis in Surgery was 21.0% (N=1598) in the pre-ASP period and 80.0% (N=841) in the post-ASP period (59% absolute improvement, p < 0.001). Percentage of optimal duration was 69,1% (N=1598) in the pre-ASP period and 78.0% (N=841) in the post-ASP period (8.9% absolute improvement, p < 0.001). Mean ceftriaxone utilization was 217.7 defined daily doses (DDD) per 1,000 patient days DDD for the pre-ASP period and 139.8 DDD per 1,000 patient days for the ASP period (35.8% decrease; p = 0.019). Mean cefazolin utilization was 14.9 DDD per 1,000 patient days for the pre-ASP period and 153.3 DDD per 1,000 patient days for the ASP period (928.6% increase; p = 0.021). Regarding percentage of bacterial resistance, there was detected an improvement in some isolates like *Escherichia coli* with a decrease of ESBL detection (11% decrease; p = 0.007). In addition, no serious adverse reactions or an increase in surgical site infections were detected after the intervention.

**Conclusion:**

The implementation of an ASP in the surgical ward showed an overall positive impact on selection and duration of antibiotic prophylaxis. Furthermore, this intervention could have had a positive impact on antimicrobial resistance and at the same time had no negative effects on the patients.

**Disclosures:**

**All Authors**: No reported disclosures

